# Aggregatibacter actinomycetemcomitans Causing Empyema Necessitans and Pyomyositis in an Immunocompetent Patient

**DOI:** 10.7759/cureus.9454

**Published:** 2020-07-29

**Authors:** Nora Homsi, Rajendra Kapila

**Affiliations:** 1 Infectious Diseases, Rutgers New Jersey Medical School/Trinitas Regional Medical Center, Newark, USA

**Keywords:** empyema necessitans, empyema necessitatis, aggregatibacter actinomycetemcomitans, immunocompetent, pyomyositis

## Abstract

Empyema necessitans is a relatively rare clinical entity in which the empyema extends through the parietal pleura into the adjacent soft tissue and musculature of the chest wall. It usually occurs due to inadequate treatment of a primary lung infection. Aggregatibacter (formerly Actinobacillus) actinomycetemcomitans is a facultative anaerobic gram-negative coccobacillus that is part of the normal oral flora. Infections due to this organism usually result from aspiration in conjunction with dental disease or trauma to the oral mucosa resulting in pneumonia or empyema. It often coinfects with Actinomyces and is known to cause empyema necessitans. Cases of monomicrobial empyema necessitans due to Aggregatibacter actinomycetemcomitans in adults have rarely been reported with four such publications found on review of the literature. We present a patient with severe periodontitis who developed empyema necessitans due to Aggregatibacter actinomycetemcomitans likely from aspiration complicated by pyomyositis of the right triceps brachii and a left posterior thigh abscess.

## Introduction

Empyema necessitans, a rare complication of empyema, is a pyogenic infection extending directly from the pleural cavity into the overlying subcutaneous tissue and causes infection in surrounding structures, such as the chest wall, mediastinum, pericardium, esophagus, and retroperitoneum [1,2]. Several causative pathogens have been reported in the literature and include Mycobacterium tuberculosis, Streptococcus pneumoniae, Staphylococcus aureus (including methicillin-resistant Staphylococcus aureus [MRSA]), Pseudomonas, Actinomyces, and others [1-4].

We present a patient who developed empyema necessitans due to Aggregatibacter (formerly Actinobacillus) actinomycetemcomitans likely from aspiration in the setting of periodontitis. His infection was complicated by pyomyositis of the right triceps brachii and a left posterior thigh abscess. Review of the literature revealed four previous publications of patients with monomicrobial empyema necessitans due to Aggregatibacter actinomycetemcomitans [5-8].

## Case presentation

**Figure 1:**
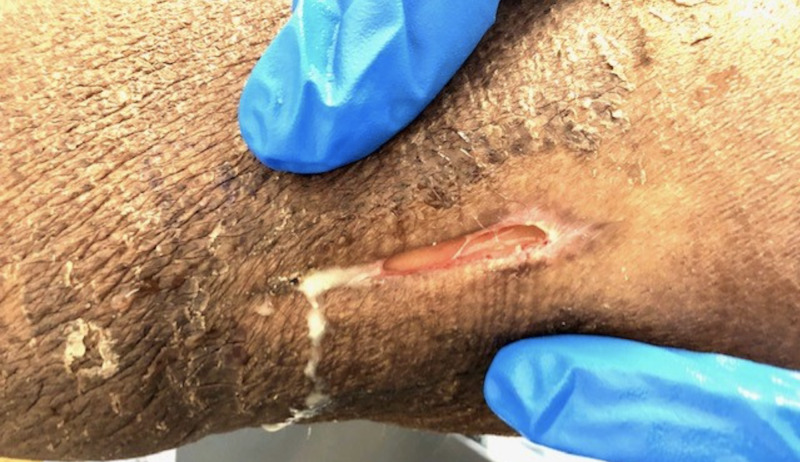
Musculocutaneous fistula on posterior surface of right upper arm with scant purulent drainage.

A 57-year-old gentleman with severe periodontitis presented with pyomyositis of the right triceps brachii as seen on CT scan and underwent incision and drainage. Intraoperative cultures taken from the intramuscular abscess grew Aggregatibacter actinomycetemcomitans. He received four doses of intravenous vancomycin 1.25 g over a period of three days and was discharged home without oral antibiotics but with outpatient follow-up with the surgeon. The infectious diseases team was not consulted at that time. Two months later, he presented again to our hospital with a painful and erythematous right upper chest wall mass that had been enlarging over the past two weeks and was associated with subjective fevers and a nonproductive cough. He denied any trauma to the site. He also complained of a bad taste in his mouth that was limiting his oral intake. He denied a smoking history or use of illicit drugs or alcohol. On physical examination, he was febrile (101.6˚F) with the remainder of his vital signs within normal limits. He had severe gingivitis and multiple dental caries in the few teeth he had remaining. There was a 5-cm tender, erythematous, and fluctuant mass on his right upper chest wall, and rales were heard in the right lung field. There was a musculocutaneous fistula on the posterior surface of right upper arm that tracked up his arm with scant purulent drainage (Figure 1). Cardiac and abdominal exams were benign. Examination of his lower extremities at the time of presentation did not reveal any abnormalities.

Laboratory studies were significant for leukocytosis of 20.7 x 10^3 ^cells/μL with bandemia of 19%, anemia with hemoglobin of 8.1 g/dL, a reactive thrombocytosis of 664 x 10^3 ^platelets/μL, and elevated C-reactive protein (CRP) of 248 mg/L and erythrocyte sedimentation rate (ESR) of 119 mm/hr. Kidney and liver function were within normal limits. A fourth-generation HIV screen and a hepatitis panel were nonreactive, and four sets of blood cultures were negative.

**Figure 2:**
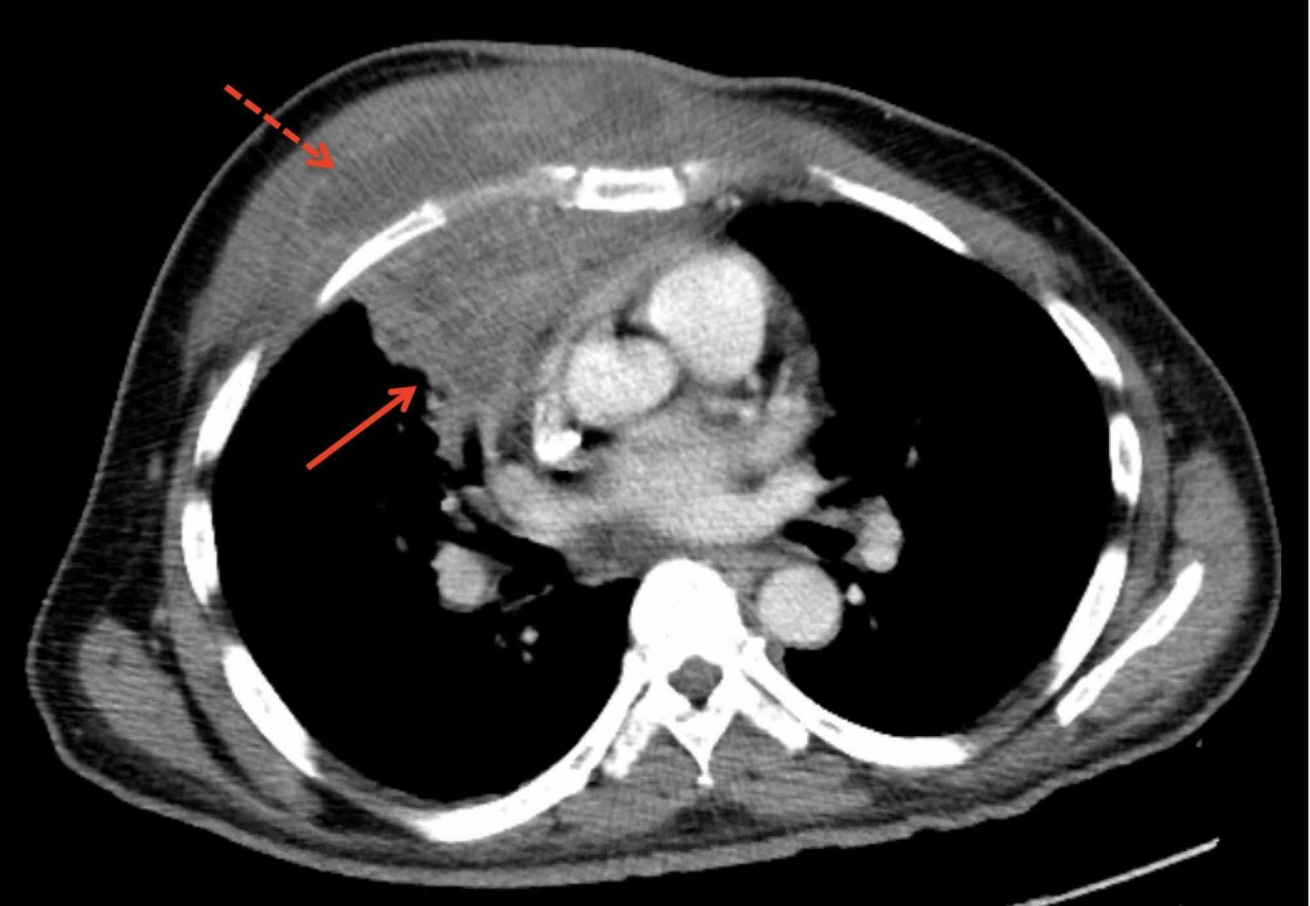
CT with contrast of the chest showing an abscess in the right lung (solid arrow) with extension through the pleura and into the soft tissue and musculature of the right chest wall (dashed arrow).

CT scan with contrast of the chest revealed a right intra- and extrathoracic abscess with involvement of the right pectoralis muscle, pleura, and right upper and middle lobes (Figure 2). Transthoracic echocardiography did not show evidence of infective endocarditis. Review of the CT scan of his right upper extremity from two months earlier revealed a partially seen right lung consolidation that had not been previously reported, suggesting a pulmonary origin of infection. In light of his severe dental disease, aspiration was most likely the precipitating event.

**Figure 3:**
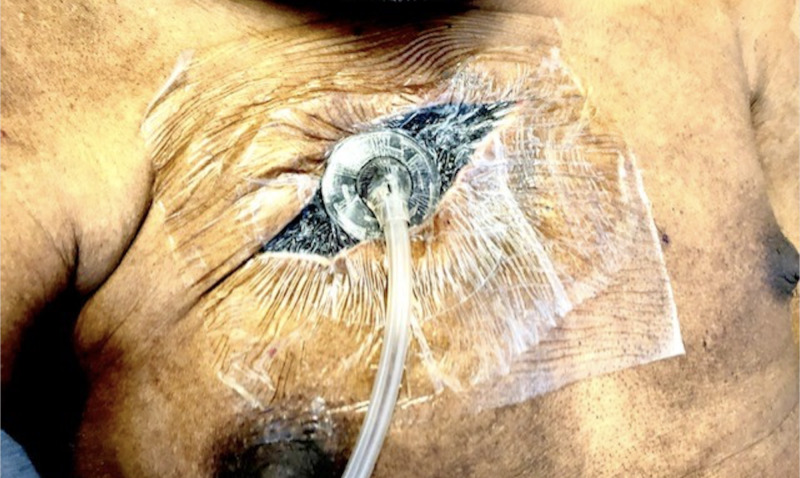
Vacuum-assisted closure of wound of right upper chest wall following the initial surgical debridement.

He underwent debridement of the right chest abscess with partial resection of his right second rib (Figure 3) followed by multiple washouts and finally a grafting procedure. Cultures taken from the abscess in the operative room during the first debridement grew Aggregatibacter actinomycetemcomitans (beta-lactamase negative), the same organism that had grown from the right triceps brachii abscess. One week into his admission, he was noted to have a new left posterior thigh abscess that was also debrided. The infectious diseases team was consulted for antibiotic recommendations during his second admission. He received empiric therapy with intravenous vancomycin 1.25 g every 12 hours and intravenous piperacillin-tazobactam 4.5 g every 6 hours for 14 days followed by intravenous ceftriaxone 2 g daily for 10 more days once wound cultures were finalized. Upon discharge, he was transitioned to oral amoxicillin-clavulanate 875-125 mg every 12 hours to complete six weeks of treatment from his last debridement. On outpatient follow-up two months after discharge, he remained infection-free with normalization of his acute phase reactants.

## Discussion

Empyema necessitans is a relatively rare clinical entity in which the empyema extends through the parietal pleura into the adjacent soft tissue and musculature of the chest wall. It usually occurs due to inadequate treatment of a primary lung infection [1,2]. Several causative pathogens have been reported in the literature and include Mycobacterium tuberculosis, Streptococcus pneumoniae, Staphylococcus aureus (including MRSA), Pseudomonas, Actinomyces, and others [1-4]. Treatment involves surgical debridement of infected tissue in combination with antibiotics tailored to the pathogen [1].

Aggregatibacter (formerly Actinobacillus) actinomycetemcomitans is a slow-growing facultative anaerobic gram-negative coccobacillus [5-7]. Part of the normal oral flora, it frequently plays a pathogenic role in periodontal disease [5,6,8,9]. It has also been implicated as a causative agent in infective endocarditis, soft tissue abscesses, pneumonia, and osteomyelitis [6-9]. Infection occurs via hematogenous dissemination or aspiration into the respiratory tract with direct invasion of surrounding structures in the setting of dental disease [5,6,7,9]. It often causes coninfection with Actinomyces and is known to cause empyema necessitans [6,8-10].

Cases of monomicrobial empyema necessitans due to Aggregatibacter actinomycetemcomitans in adults have rarely been reported [5-8]. Table 1 summarizes the four previously reported patients. Three were men and one was a woman with ages ranging from 50 to 67 years. All were immunocompetent. Three were diagnosed with pneumonia and chest wall masses. One patient had a mediastinal abscess with an overlying chest wall mass without a reported pneumonia. Two patients had some degree of dental disease, one patient was a smoker, and the fourth patient did not have any predisposing factors. All four patients received antibiotic therapy with beta-lactams with duration ranging from four weeks to 12 months. Two patients underwent surgical excision in addition to antibiotic therapy. All patients fully recovered.

**Table 1:** Previously published reports of patient with monomicrobial chest wall infections due to Aggregatibacter actinomycetemcomitans. Abd=abdominal; AMP=ampicillin; AMX=amoxicillin; CLV=calvulanate; CTX=ceftriaxone; F=female; IV=intravenous; M=male; PCN=penicillin; PNA=pneumonia; PO=oral; SBT=sulbactam *Year infection and treatment occurred; does not necessarily correlate with year article was published

Age (years)/sex	Year^*^	Immune status	Predisposing factor(s)	Infection	Surgical treatment	Antibiotics/duration	Outcome	Reference
50/M	1982	Immunocompetent	None	Mediastinal abscess, chest wall mass	Excision, drainage	PO AMX/4 months	Full recovery	[5]
67/M	1990	Immunocompetent	Marginal gingivitis	PNA, chest wall mass with rib destruction	None	IV PCN G, PO AMX/3 months	Full recovery	[6]
56/F	2004	Immunocompetent	Dental caries	PNA, chest wall mass	Excision	IV AMP-SBT, PO AMX-CLV/4 weeks	Full recovery	[7]
54/M	2017	Immunocompetent	Smoker	PNA, chest and abd wall masses	None	IV CTX, PO AMX/12 months	Full recovery	[8]

There is no optimal antibiotic therapy for Aggregatibacter actinomycetemcomitans. It usually demonstrates in vitro susceptibility to cephalosporins, aminoglycosides, fluoroquinolones, and tetracyclines; it has variable susceptibility to penicillin and ampicillin [6,7]. It has frequently been reported to be resistant to vancomycin and clindamycin. Previously published case reports have shown successful treatment of invasive infection caused by this pathogen with intravenous ceftriaxone or penicillin followed by oral amoxicillin or with oral amoxicillin alone [5-8,10]. It is recommended to obtain susceptibility testing in order to better guide antibiotic selection. Treatment duration is dependent on clinical course, but extended courses are often necessary [6,7].

## Conclusions

This case highlights the importance of determining the primary site of a disseminated infection. The patient initially presented with a pyomyositis of the right triceps brachii with no evidence of trauma or inciting event, and abscess cultures grew an unusual pathogen, Aggregatibacter actinomycetemcomitans. In such a situation, physicians should cogitate and further evaluate to determine the primary site of infection. In some cases, early discussion with an infectious diseases specialist may be warranted.

During the patient’s first admission, the pulmonary consolidations seen on the CT of the right upper extremity were overlooked. They were later confirmed with a chest CT only after his old imaging was reviewed during his second admission, resulting in delayed diagnosis. Thus, another important lesson that this case emphasizes is the need for all clinicians to personally review the images pertaining to their patients while maintaining communication with radiologists.
